# Torque and Velocity Dependence of Muscle Fatigue in Aging

**DOI:** 10.1093/geroni/igaa057.1590

**Published:** 2020-12-16

**Authors:** Liam Fitzgerald, Anthony Martin, Rajakumar Nagarajan, Jane Kent

**Affiliations:** 1 University of Florida; 2 University of Massachusetts Amherst, Amherst, United States; 3 University of Massachusetts Amherst, Amherst, Massachusetts, United States

## Abstract

Old age generally leads to smaller, weaker and slower skeletal muscles. To address the independent effects of weakness vs. slowing on fatigue in aging, we used a custom ergometer in a whole-body, 3 tesla magnetic resonance system to quantify knee extensor size, torque, velocity, power and intracellular energetics at baseline and during two 4-min fatiguing contraction protocols; one in which contraction velocity was constrained and torque varied (i.e., torque-dependent contractions; isokinetic, IsoK), and one in which torque was constrained and velocity varied (i.e., velocity-dependent contractions; isotonic, IsoT). On separate days, 10 young (27.5±1.2 yrs, 6 men) and 10 older (71.2±1.6 yrs, 5 men) healthy adults completed the IsoK (120°∙s-1, 0.5 Hz) and IsoT (20% maximal torque, 0.5 Hz) protocols, with continuous measures of intracellular [Pi], pH, and [H2PO4-]. At baseline, contractile volume (803.5±72.3 vs. 1,125.6±109.9cm3), specific IsoK torque (0.035±0.004 vs. 0.058±0.007Nm.cm-3) and IsoT velocity (121.4±11.6 vs. 176.3±8.0deg.s-1) were greater in young than older (p≤0.023). Fatigue (%initial specific torque) was greater in young than older for IsoK (40.1±3.0 vs. 61.2±5.3%, p=0.0028), and accompanied by greater [Pi] and [H2PO4-] and lower pH in the young (p≤0.001). For IsoT, fatigue (%initial velocity) was not different between groups (young: 56.5±5.5 vs. older: 47.2±4.9%, p=0.661), despite lower pH and greater [H2PO4-] in young than old (p≤0.001). Collectively, these results reveal that normalizing dynamometer outputs to assess age-related differences in fatigue obscures baseline differences in muscle weakness. Further, our results suggest the contractile machinery may be less sensitive to changes in pH in older than young.

